# Osteoporosis care in primary care settings: a national UK e-survey

**DOI:** 10.1007/s11657-025-01591-8

**Published:** 2025-08-06

**Authors:** Ashley Hawarden, Laurna Bullock, Natasha Marie Cox, Elaine Nicholls, Jo Protheroe, Clare Jinks, Zoe Paskins

**Affiliations:** 1 Primary Care Centre Versus Arthritis, Keele University, Keele, Staffordshire UK; 2 Haywood Academic Rheumatology Centre, Haywood Hospital, Midlands Partnership University NHS Foundation Trust, High Lane, Burslem, Staffordshire UK; 3 Keele Clinical Trials Unit, Keele University, Keele, Staffordshire UK

**Keywords:** Osteoporosis, Primary care, Survey

## Abstract

**Summary:**

An electronic survey of 341 UK primary care staff identified barriers to evidence-based osteoporosis care including low confidence in clinical skills, the complex nature of decision-making, insufficient incentivisation and lack of systematic case finding. Opportunities to enhance osteoporosis care may include enhanced education and wider utilisation of the extended workforce.

**Purpose:**

To investigate the beliefs, confidence and practices of general practice staff in the care of people with, or at increased risk of, osteoporotic fractures and the association between professional role and beliefs and confidence about osteoporosis care.

**Methods:**

An electronic survey was designed and distributed to UK general practice staff, including healthcare professionals (HCPs) and non-healthcare professionals (non-HCPs). Content was informed by UK clinical guidelines, a scoping review and patient and clinical stakeholder input. Descriptive statistics and Fisher’s exact test were utilised for analysis, with free text responses analysed using reflexive thematic analysis.

**Results:**

Three hundred forty-one responses were obtained (309 HCPs, 32 non-HCPs). Most responding HCPs (173, 62.2%) and non-HCPs (17, 70.8%) reported osteoporosis management of moderate priority. The majority of HCPs (228, 73.8%) agreed that they were worried about osteoporosis medicines causing unpleasant side effects. Most respondents (314, 98.7%) reported GPs as involved in osteoporosis care, followed by Pharmacists (241, 75.8%) and Practice Nurses (159, 50.0%). GPs and Pharmacists reported the highest level of agreement with confidence in osteoporosis medicine related skills. Fewer than a third of respondents reported systematic invitation of patients with risk factors (fracture, steroids or falls) for assessment. Free text responses indicated problems with communication between primary and secondary care, challenging decision-making, limited access to resources (e.g. DXA scan, dentistry) and insufficient incentivisation as barriers to delivery of recommended osteoporosis care.

**Conclusion:**

Identified opportunities to improve osteoporosis care include improved education, incentivisation, automated case finding and involvement of the wider primary care workforce, particularly Pharmacists.

**Supplementary Information:**

The online version contains supplementary material available at 10.1007/s11657-025-01591-8.

## Introduction

In the United Kingdom (UK), primary care serves as the “front door” of the National Health Service (NHS), providing the primary point of access to healthcare services and includes general practice, community pharmacy and dental services. As such, general practice plays a crucial role in the identification, investigation and treatment of people with, or at risk of, osteoporotic fracture. Effective primary care management of osteoporosis is essential given the burden of osteoporotic fractures on the NHS. There are over 500,000 osteoporotic fractures annually in the UK and by 2030 the number is predicted to rise by 26% [1, 2].

To reduce fracture incidence, international guidance recommends systematic identification of people with clinical risk factors, followed by bone health assessment, and for those at increased risk, intervention with cost and clinically effective fracture risk-reducing strategies, including osteoporosis medicines [3, 4]. However, a worldwide osteoporosis treatment gap, defined as “the ‘gap’ between those individuals who require treatment and those individuals who actually receive treatment” exists [5, 6]. The treatment gap in Europe for women aged ≥ 70 and at risk of fragility is estimated to be 74.6% [7].

In the UK, general practice plays a role in both primary and secondary fracture prevention. While secondary prevention is often led by Fracture Liaison Services (FLSs) in secondary care, general practice retains an important role. Currently, FLSs cover only 51% of NHS Trusts and, despite best efforts, do not identify all at-risk individuals [8]. Additionally, a third of FLSs do not discuss osteoporosis medications with patients, and most do not prescribe them, underscoring the need for close collaboration with general practice [9].

International survey evidence suggests that general practitioners report variable utilisation of osteoporosis guidelines [10–12], have limited access to dual-energy x-ray absorptiometry (DXA) [11, 13, 14], do not participate in systematic screening [13] and have notable knowledge gaps, particularly relating to pharmacotherapy [13–16]. However, little is known about the routine practice of osteoporosis care in UK general practice. The only previous comprehensive survey of osteoporosis care in UK general practice is over 20 years old [17]. A contemporaneous perspective is needed, due to both the changes in osteoporosis management over the last two decades, and the transformation of the primary care workforce in the UK. Traditionally, GPs have been the cornerstone of specialist care delivery in general practice; however, the scope of practice of other members of the multidisciplinary team has expanded significantly in recent years, including Advanced Nurse Practitioners, practice-based Pharmacists and First Contact Physiotherapy Practitioners as examples [18].

This national electronic survey (e-survey) aims to investigate the beliefs, confidence in management and current routine practices of general practice staff in the care of people with, or at increased risk of, osteoporotic fracture. Additionally, this e-survey aimed to investigate the association between professional role and beliefs and confidence about osteoporosis and osteoporosis care.

## Methods

An e-survey was designed and distributed to UK general practice staff, including healthcare professionals (HCPs) and non-HCPs such as managerial staff.

### Survey design and content

The e-survey was hosted using a web-based survey platform (HealthSurvey, using secure servers at Keele University). The e-survey content was informed by:

- UK clinical guidelines [19–23]
- A scoping review exploring the enablers and barriers to the provision of osteoporosis care in primary care settings [24] and previous surveys of musculoskeletal conditions in primary care [15, 25]
- Validated questionnaires (used previously in UK populations) that aim to understand people’s beliefs about illness [26] and beliefs about medicines [27].
- Feedback from two Osteoporosis Research User Group meetings: The first meeting (with seven public contributors) focused on survey content, design and recruitment strategies, while the second meeting (with five public contributors) provided input on the analysis plan.
- One meeting of the Keele Osteoporosis Community of Practice members comprising a multidisciplinary group (*n* = 16) of UK-based primary (GPs (*n* = 5), Advanced Nurse Practitioner (*n* = 1), Physiotherapy First Contact Practitioner (*n* = 1), Clinical Pharmacist (*n* = 1)) and secondary (rheumatologists (*n* = 2), radiographer (*n* = 1), FLS Nurse Specialist (*n* = 1)) care clinicians, alongside representatives from charity (*n* = 1, Royal Osteoporosis Society), industry (*n* = 1, UCBPharma) and individuals with lived experience of osteoporosis (*n* = 2).

E-survey questions were filtered according to participant’s self-reported professional role. The complete survey consisted of nine sections:

1. *Professional primary role*.
2. *Beliefs about osteoporosis and osteoporosis care*: captured using Likert scale responses.
3. *Confidence in osteoporosis care*: captured using Likert scale responses *[shown only to HCPs]*.
4. *Professional roles and responsibilities*: captured perceptions of the healthcare practitioners involved in the delivery of osteoporosis care, as well as the roles they perform.
5. *Experience of delivering osteoporosis care*: including investigation, information giving and management *[shown only to HCPs]*.
6. *Challenges in osteoporosis care*: explored perceived knowledge of osteoporosis and opportunities for improvement in osteoporosis care *[shown only to HCPs]*.
7. *Osteoporosis care at the practice level*: captured the provision of osteoporosis care in the respondent’s primary care setting with reference to national osteoporosis guidance.
8. *Participant demographics*: including age, sex, ethnic group (defined using the Office for National Statistics Census 2021 five high-level ethnic groups [28]) and location of primary care service.
9. *Comments and suggestions*: allowed respondents to provide free text response to the question: “Are there any other comments or suggestions you would like to make about the care of people with, or at risk of, osteoporosis in primary care settings?”

The e-survey was piloted by the study team (JP – Academic GP, ZP—Academic Rheumatologist, CJ – Applied Health Researcher), one GP and a Practice Nurse. The e-survey was updated in response to their feedback. Updates included e-survey formatting and question logic rather than question content.

### Patient and public involvement

Public contributors were involved in the design, analysis and interpretation of the e-survey via the osteoporosis Research User Group and as members of the Community of Practice. The aim of patient and public involvement was to identify and prioritise areas of osteoporosis care most important to those with lived experience. Involvement has been reported with reference to Guidance for Reporting Involvement of Patients and the Public (version 2, Short Form) [29]. Public contributors specifically advised on survey incentivisation (option for charitable donation), recruitment (inclusion of non-GP primary care staff), survey content (e.g. assessment of patient information resources) and analysis (e.g. comparison of results based on job role). Additionally, public contributors supported the inclusion and analysis of free text comments to capture nuanced insights beyond the quantitative data.

### Participants and recruitment

Primary care staff, regardless of job role, from across the UK were eligible to participate in the e-survey. The survey was disseminated through multiple channels, including:

- Local Clinical Research Networks in England, Health and Care Research Wales and NHS Research Scotland
- X, formally known as Twitter with re-tweets from relevant charities, societies and organisations including Versus Arthritis (38.6 k followers), Primary Care Rheumatology and Musculoskeletal Medicine Society (3.6 k followers), the Royal Osteoporosis Society (9.6 k followers), Health and Care Research Wales (7.5 k followers), NIHR Research Delivery Network (8 k followers), Keele Impact Accelerator Unit (1.2 k followers) and NHS Research Scotland (9 k followers)
- Clinical and academic networks of the study team to facilitate onward dissemination.

To incentivise participation, a prize draw was offered, allowing participants to win one of three £100 shopping vouchers or donation to charity. Non-monetary incentives, such as prize draws, have been shown to double the odds of survey response [30].

### Sample size and data analysis

Using the Clopper-Pearson exact method and an estimated proportion of 0.5, a sample size of 200 was determined a priori to estimate proportions with a precision of ± 0.14 or less at a 95% confidence level. Survey data were analysed using StataMP. Descriptive statistics summarised the data using frequencies and proportions. Likert scale responses exploring participants’ beliefs and confidence about osteoporosis were dichotomised into two categories: “agree” (consisting of “strongly agree” and “agree”) and “do not agree” (consisting of “neither agree or disagree”, disagree and “strongly disagree”). Where there were more than ten e-survey respondents per job role (GPs, Practice Nurses, Pharmacists and Advanced Nurse Practitioners), the association between job role and agreement to statements for beliefs about osteoporosis (survey Section 2) and confidence in delivering osteoporosis care (survey Section 3) were examined using Fisher’s exact test (used rather than chi-square due to sample sizes of ≤ 5 [31]).

Free text response were conceptualised as an a priori adjunct to the quantitative analysis and analysed using reflexive thematic analysis [32–34]. This approach was chosen for its theoretical flexibility and capacity to capture nuanced perspectives. A six-phase approach to analysis was taken, facilitated by NVivo 14, which supported data organisation, semantic coding and theme development [30]. The six phases included (i) initial immersion in the data; (ii) application of code labels; (iii) generation of initial themes; (iv) development and review of themes; (v) refinement, defining and naming of themes; and (vi) the production of a report.

AH, an academic rheumatologist with a specialist interest in metabolic bone disease, conducted the analysis. Recognising the potential for bias due to his professional background, AH engaged in iterative reflexivity throughout the analysis by writing reflexive memos during analysis and critically discussing data interpretations with public contributors and study team members (ZP and CJ) to unpick how his assumptions and expertise shaped his understanding of the data.

### Ethical review

Favourable ethical opinion was provided by the Keele University Research Ethics Committee (reference 0359) and Health Research Authority (HRA) and Care Research Wales approval was granted (reference 23/HRA/2027). The participant information sheet and consent form were embedded in the e-survey and participants were not able to proceed without providing informed consent.

## Results

The e-survey link was active for 10 weeks, from August to October 2023. A total of 341 responses were obtained (314 complete and 27 partial responses), including contributions from 309 HCPs and 32 non-HCPs.

Respondent characteristics are summarised in Table 1; most were female (201, 63.6%), self-reported as being of white heritage (256, 81.5%) and worked in general practices in England (290, 91.77%). The most common role was GP (198, 64.1%) for HCPs, and Manager (20, 62.5%) for non-HCPs.

**Table 1:** Baseline demographic details of respondents

Characteristic	Response	Total	HCPs	Non-HCPs
		*n* (%)	*n* (%)	*n* (%)
**Sex**^**a**^ ^***n* =total (316), HCPs (288), non−HCPs (28)**^	Female	201 (63.6)	178 (61.8)	23 (82.1)
	Male	112 (35.4)	107 (37.2)	5 (17.9)
	Prefer not to say	3 (1.0)	3 (1.0)	0 (0.0)
**Age**^**a**^ ^***n* =total (316), HCPs (288), non−HCPs (28)**^	20 years or younger	1 (0.3)	0 (0.0)	1 (3.6)
	21–30 years	18 (5.7)	12 (4.2)	6 (21.4)
	31–40 years	96 (30.4)	93 (32.3)	3 (10.7)
	41–50 years	105 (33.2)	101 (35.1)	4 (14.3)
	51–60 years	72 (22.8)	64 (22.2)	8 (28.6)
	61–70 years	21 (6.7)	15 (5.2)	6 (21.4)
	71–80 years	1 (0.3)	1 (0.4)	0 (0.0)
	Prefer not to say	2 (0.6)	2 (0.7)	0 (0.0)
**Ethnic group**^**a,b**^ ^***n* =total (314), HCPs (287), non−HCPs (27)**^	Asian, Asian British, Asian Welsh	44 (14.0)	43 (15.0)	1 (3.7)
	Black, Black British, Black Welsh, Caribbean or African	4 (1.3)	3 (1.1)	1 (3.7)
	Mixed or Multiple ethnic groups	5 (1.6)	5 (1.7)	0 (0.0)
	White	256 (81.5)	231 (80.5)	25 (92.6)
	Other ethnic group	5 (1.6)	5 (1.7)	0 (0.0)
	Prefer not to say	0 (0.0)	0 (0.0)	0 (0.0)
**Country**^**a**^ ^***n* =total (316), HCPs (288), non−HCPs (28)**^	England	290 (91.8)	262 (91.0)	28 (100)
	Scotland	11 (3.5)	11 (3.8)	0 (0.0)
	Wales	13 (4.1)	13 (4.5)	0 (0.0)
	Northern Ireland	2 (0.6)	2 (0.7)	0 (0.0)
	Prefer not to say	0 (0.0)	0 (0.0)	0 (0.0)
**Role** ^***n* =total (341), HCPs (309), non−HCPs (32)**^	GP	198 (58.1)	198 (64.1)	0 (0.0)
	Practice Nurse	29 (8.5)	29 (9.4)	0 (0.0)
	Advanced Nurse Practitioner	27 (7.9)	27 (8.7)	0 (0.0)
	Manager	20 (5.87)	0 (0.0)	20 (62.5)
	Pharmacist	15 (4.4)	15 (4.9)	0 (0.0)
	Administrative Staff	12 (3.5)	0 (0)	12 (37.5)
	Other HCP role	11 (3.2)	11 (3.6)	0 (0.0)
	Resident Doctor	10 (2.9)	10 (3.3)	0 (0.0)
	First Contact Practitioner	6 (1.8)	6 (1.9)	0 (0.0)
	Physiotherapist	5 (1.5)	5 (1.6)	0 (0.0)
	Paramedic	4 (1.2)	4 (1.3)	0 (0.0)
	Research Nurse	4 (1.2)	4 (1.3)	0 (0.0)

^a^Demographic data (other than job role) was collected at the end of the e-survey, which likely contributed to participant attrition and a higher non-response

^b^Ethnic group categories were derived from the UK census 2021 [28]

Results of the e-survey are presented in seven parts: beliefs about osteoporosis care, confidence in delivering care, individual-level care, professional roles and responsibilities, practice-level care, challenges and free text responses.

### Beliefs about osteoporosis and osteoporosis care

The majority of responding HCPs (173, 62.2%) and non-HCPs (17, 70.8%) reported osteoporosis management to be of moderate priority in primary care settings. Fewer considered it to be of high (56, 20.1% HCPs and 4, 16.7% non-HCPs), and low (47, 16.9% HCPs and 3, 12.5% non-HCPs).

**Fig. 1:**
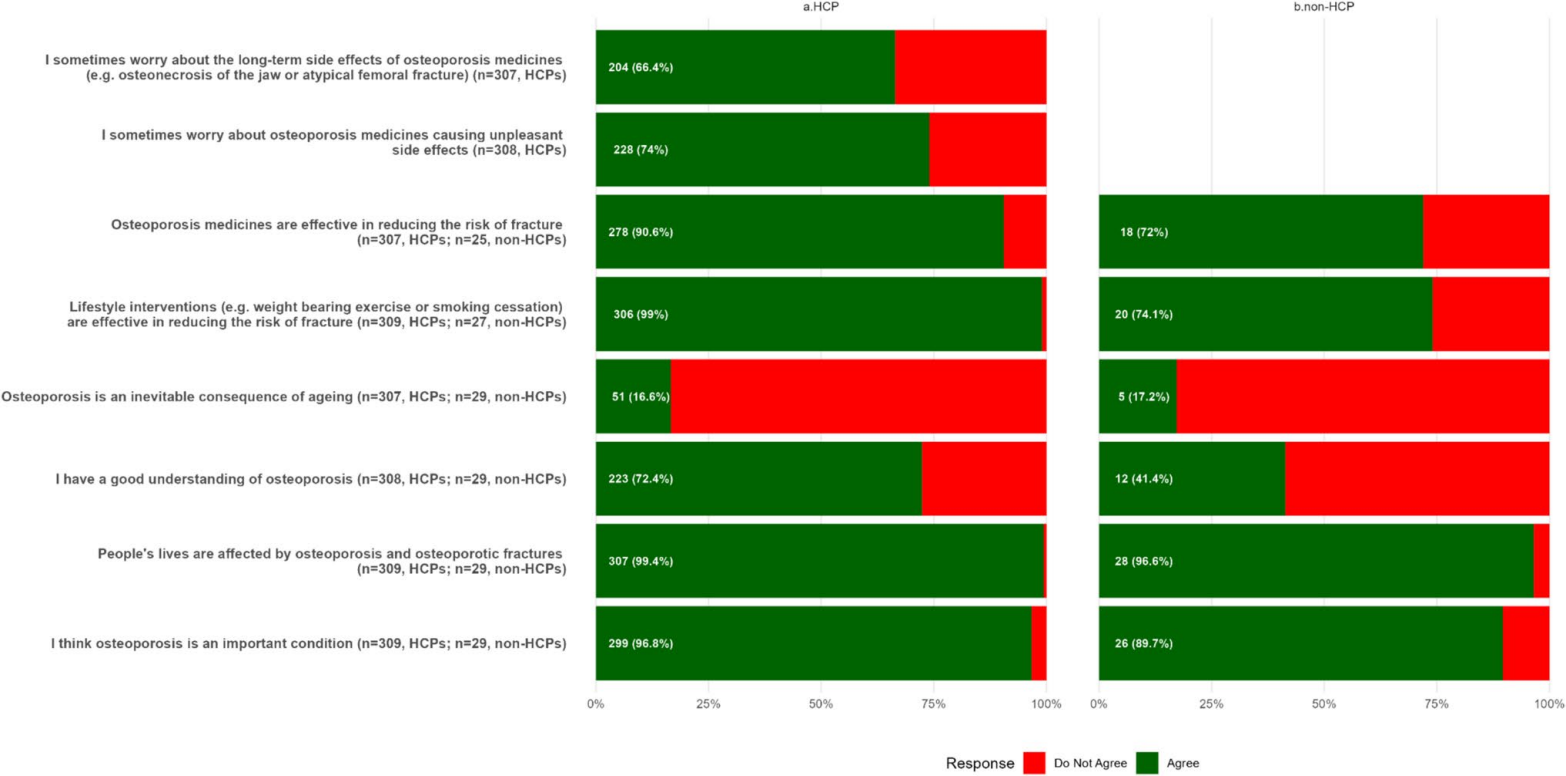
HCP and non-HCP agreement and non-agreement with statements designed to elicit individual beliefs about osteoporosis

Results regarding individual beliefs about osteoporosis for HCPs and non-HCPs are shown in Fig. 1 and Supplementary Data 2. Most HCPs agreed that osteoporosis is an important condition (299, 96.8%), people’s lives are affected by osteoporosis and osteoporotic fractures (307, 99.4%) and 256 (83.4%) did not agree that osteoporosis is an inevitable consequence of aging. Similar views were expressed by non-HCPs, although fewer reported having a good understanding of osteoporosis (HCPs 223, 72.4% vs non-HCPs 17, 58.6%). Proportionally more HCPs than non-HCPs agreed that lifestyle interventions (HCPs 306, 99.0% vs non-HCPs 20, 20, 74.1%) and osteoporosis medicines are effective at reducing fracture risk (HCPs 278, 90.6% vs non-HCPs 18, 72.0%). However, 228 (73.8%) of responding HCPs agreed that they worried about osteoporosis medicines causing unpleasant side effects, and 204 (66.5%) were worried about long-term side effects; non-HCPs were not asked to respond to medication side effects questions.

When examining differences between participants with different professional roles (Supplementary Data 2), proportionally more GPs (161, 81.3%), than Practice Nurses (12, 42.9%) and Advanced Nurse Practitioners (13, 48.1%), agreed they had a good understanding of osteoporosis (*P* < 0.001). Furthermore, compared to GPs (183, 93.4%), significantly fewer Practice Nurses (22, 75.9%) agreed that osteoporosis medications are effective in reducing fracture risk (*P* = 0.007). More GPs had concerns about the side effects of osteoporosis medications than Practice Nurses and Advanced Nurse Practitioners, with significantly more GPs than Practice Nurses expressing concerns regarding unpleasant (GPs 160, 81.2% and Practice Nurses 15, 51.7%) and long-term (GPs 144, 73.1% and Practice Nurses 11, 39.3%) side effects (*P* = 0.001).

### Confidence in delivering osteoporosis care

**Fig. 2:**
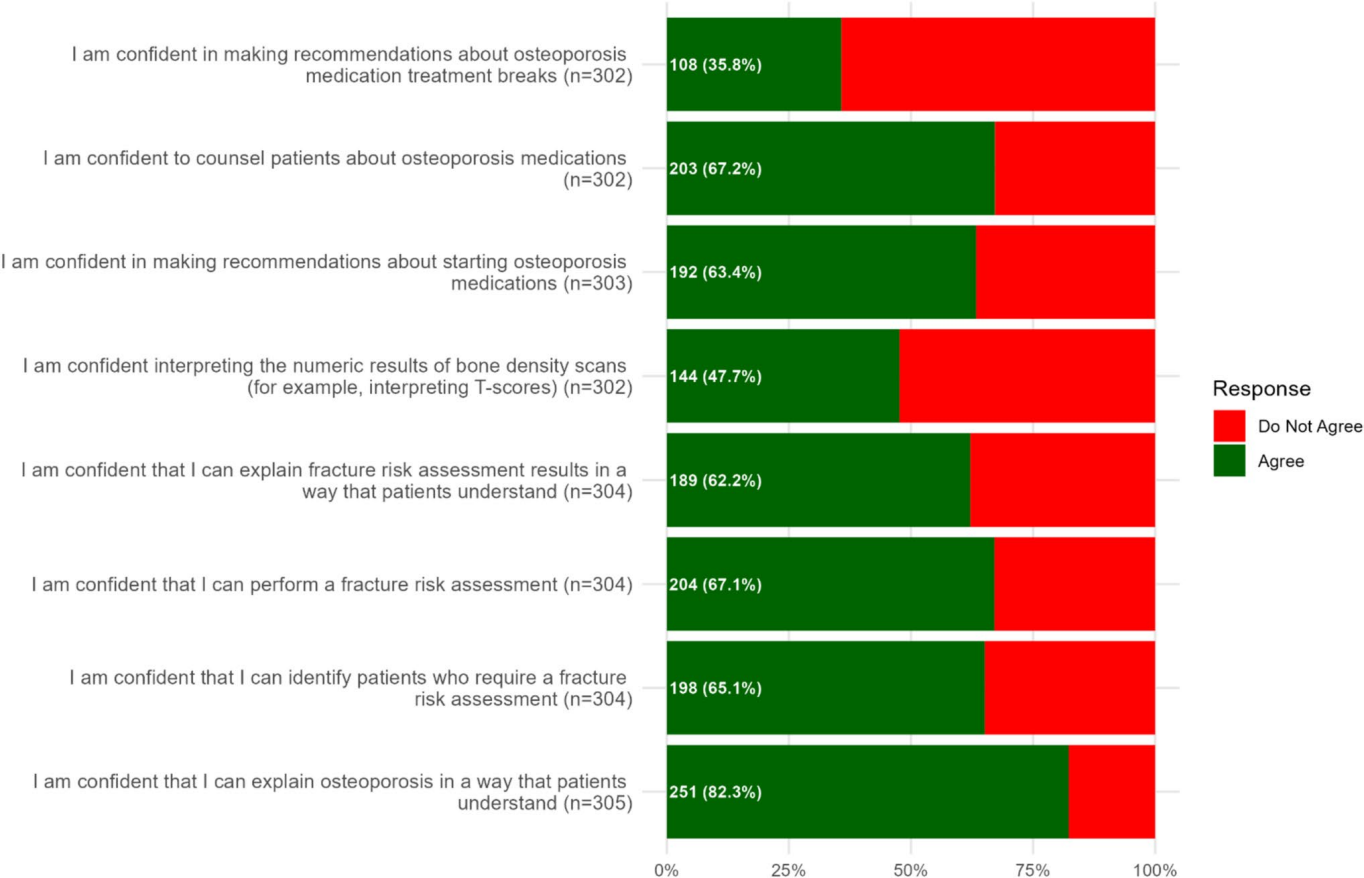
HCP agreement and non-agreement with statements designed to elicit confidence in osteoporosis management

The majority of HCPs agreed that they were confident to explain osteoporosis in a way that patients understand (251, 82.3%), identify patients who require a fracture risk assessment (198, 65.1%), perform a fracture risk assessment (204, 67.1%), explain fracture risk in a way that patients understand (189, 62.2%), make recommendations about starting osteoporosis medicines (192, 63.4%) and counsel patients about osteoporosis medicines (203, 67.2%). In contrast, more than half of HCPs did not agree with statements that they were confident to interpret the numeric results of bone density scans (158, 52.3%) or to make recommendations about treatment breaks (194, 64.2%) (Fig. 2, Supplementary Data 3).

GPs and Pharmacists reported the highest level of agreement with confidence statements, while Practice Nurses consistently reported the least agreement (Supplementary Data 4). GPs were significantly more confident than both Practice Nurses and Advanced Nurse Practitioners in explaining osteoporosis to patients, identifying patients requiring fracture risk assessments, performing fracture risk assessments, interpreting bone density results, recommending osteoporosis medications and counselling patients about treatment (*P* < 0.001 for all). Notably, while agreement with confident statements between GPs and Pharmacists were comparable in most domains, Pharmacists were significantly more confident than GPs in making recommendations about treatment breaks (*P* < 0.001).

### Individual experiences of delivering osteoporosis care

Individual experiences of delivering osteoporosis care are summarised in Table 2. More than three quarters of responding HCPs reported using a fracture risk assessment tool, with most using FRAX®. A total of 247 responding HCPs identified themselves as prescribing practitioners (199, 80.6% were doctors). Most prescribers used National Institute for Health and Care Excellence guidance to make decisions about osteoporosis medicines (160, 64.8%). A total of 149 (60.3%) prescribing HCPs reported routinely providing or directing patients to information about osteoporosis medicines. The available osteoporosis medication information resources were reported to be very good (13, 8.7%), good (70, 47.0%), fair (55, 36.9%), poor (9, 6.0%) and very poor (1, 0.7%); 1 (0.7%) participant did not provide a response.

**Table 2:** Individual experiences of delivering osteoporosis care

Individual experiences of delivering osteoporosis care	
**Use of fracture risk assessment tools**	*n* (%)*
* Number of Respondents Reporting use of Fracture Risk Assessment Tools*	*n* = 229
Self-reported use of Specific Fracture Risk Assessment Tools* FRAX QFracture Garven Other	226 (98.7) 28 (12.2) 0 (0) 1 (0.4)
**Osteoporosis medicine prescribing**	
* Number of Respondents Identifying as Prescribing Practitioners*	*n* = 247
Self-reported use of Guidelines to Make Decisions About Osteoporosis Prescribing* National Institute for Health and Care Excellence Guidelines Local/Regional Guidelines National Osteoporosis Guideline Group Guidelines Other Own Practice Guidelines Scottish Intercollegiate Guidelines Network Guidelines I Do Not Refer to Guidelines	160 (64.8) 125 (50.6) 101 (44.1) 15 (6.1) 12 (4.9) 11 (4.5) 9 (3.6)
* Number of Respondents Self-reporting Routine Provision of Patient Information About Osteoporosis Medicines*	*n* = 149
Self-reported Source of Patient Information* Patient.co.uk Drug Information NHS Drug Information Royal Osteoporosis Society Drug Information Versus Arthritis Drug Information Product Specific Literature Other	83 (55.7) 83 (55.7) 45 (30.2) 40 (26.8) 33 (22.1) 7 (4.7)
* Self-reported Quality of Patient Information* Very good Good Fair Poor Very poor No response	13 (8.7) 70 (47.0) 55 (36.9) 9 (6.0) 1 (0.7) 1 (0.7)
* Number of Respondents Self-reporting No Routine Provision of Patient Information About Osteoporosis Medicines*	*n* = 98
* Number of Respondents Who Report Routine Organisation of a Medication Review After Prescribing Osteoporosis Medicines*	*n* = 131
* Self-reported Type of Medication Review Organised After Prescribing Osteoporosis Medicines* Osteoporosis Specific Medication Review Part of a Generic Medication Review No Response	33 (25.2) 97 (74.0) 1 (0.8)
* Number of Respondents Who Report No Routine Organisation of a Medication Review After Prescribing Osteoporosis Medicines*	*n* = 97
**Medication reviews**	
Number of Respondents Reporting They Conduct Osteoporosis Medication Reviews	*n* = 176
Aspects discussed during osteoporosis medication reviews* Topics Adherence to osteoporosis medications Common adverse effects of osteoporosis medications Method of taking osteoporosis medications Fracture history Alternative treatment options if adverse effects are unacceptable or the person has difficulty adhering to treatment Falls risk Rare adverse effects of osteoporosis medications Back pain Height loss Other	166 (94.3) 165 (93.8) 130 (73.9) 127 (72.2) 121 (68.8) 109 (61.9) 90 (51.1) 77 (43.8) 51 (29.0) 2 (1.1)

*Participants were not limited to the number of responses and so totals are greater than the number of respondents

Of prescribing HCPs, 131 (53.0%) reported that they routinely arrange a medication review following the prescription of an osteoporosis medication. Of those that arrange medication reviews, 97 (74.0%) stated that the review would be incorporated into a generic medication review (e.g. as part of an annual review), while 33 (25.2%) would arrange a review specifically focused on osteoporosis medicines. One participant did not specify their approach. Both generic and osteoporosis-specific medication reviews were most frequently arranged with the individual prescriber, an alternative GP or a Pharmacist. National guidance suggests checking medication tolerance at 12 to 16 weeks post prescription [20]. Approximately half (17, 51.5%) of HCPs who arrange osteoporosis-specific medication reviews would do so between 1 and 3 months.

A total of 176 HCPs reported conducting osteoporosis medication reviews. Self-reported inclusion of discussion about issues highlighted in National Institute for Health and Care Excellence quality standards for osteoporosis [18] was reasonably high apart from discussing alternative medication (121, 68.8%) and rare adverse events (90, 51.1%). Results suggest that HCPs less commonly asked about back pain (77, 43.8%) and height loss (51, 29.0%).

### Professional roles and responsibilities

Most respondents (314, 98.7%) reported GPs and Pharmacists (243, 76.4%) to be involved in osteoporosis care. In contrast, most respondents reported that Practice Nurses (159, 50.0%), Paramedics (166, 52.2%) and Healthcare Support Workers (190, 59.8%) were not involved in osteoporosis care.

A comparison of self-reported roles and the perceived roles reported by other professional groups can be seen in Supplementary Data 5. GPs self-reported near-universal involvement in tasks such as fracture risk assessment (183, 95.8%), medication counselling (189, 99.0%), case finding (164 (85.9%) and prescribing osteoporosis medications (190, 99.0%). Advanced Nurse Practitioners self-reported involvement in fracture risk assessment (15, 88.2%), falls risk assessment (17, 100%), medication reviews (12 (70.6%) and requesting blood tests for secondary causes of osteoporosis (17, 100%). Practice Nurses reported less involvement in osteoporosis care compared to GPs, Advanced Nurse Practitioners and Pharmacists, except in falls risk assessment (10, 62.5%).

Pharmacists universally reported involvement in medication counselling, prescribing and medication reviews (all 14, 100%), while involvement in case finding (7, 50.0%), requesting blood tests (7, 50.0%) and referrals for injectable therapy (9, 64.3%) were lower compared to GPs and Advanced Nurse Practitioners.

Perceived involvement of GPs, Practice Nurses, Advanced Nurse Practitioners and Pharmacists in osteoporosis tasks, as reported by other professional groups, was generally lower than the self-reported involvement. For instance, 89 (72.4%) of responding non-GPs perceived GPs as involved in case finding, compared to 164 (85.9%) self-reporting GPs. Other examples of tasks where professional groups perceived lower involvement of other groups include GPs’ involvement in medication counselling (102, 82.9% non-GPs vs 189, 99.0% self-reported); Advanced Nurse Practitioners’ role in medication reviews (52, 38.2% non- Advanced Nurse Practitioner vs 12, 70.6% self-reported); pharmacists’ role in prescribing (153, 67.4% non-pharmacists vs 14, 100% self-reported).

### Osteoporosis care at the practice and service level

Among the 289 responding HCPs and non-HCPs, access to a FLS was reported by 80 (27.7%), while 95 (32.9%) indicated their primary care service lacked access, and 114 (39.5%) were unsure.

Regarding bone density scans, a small proportion of HCPs (6, 2.1%) reported that scans can only be requested in secondary care. Most HCPs (132, 62.0%) indicated DXA scans were completed within 1–3 months of referral, in line with guidance [35]. Delays were reported by smaller proportions, including 13 (6.1%) reporting waits exceeding 12 months. In terms of adherence to guidance about DXA reporting [35], among HCPs who could request bone density scans, 132 (62.0%) reported typically receiving the report within 3 weeks of the scan and 155 (72.8%) noted that these reports typically included management advice and/or treatment recommendations.

Among the 310 responding HCPs and non-HCPs, 133 (42.9%) reported their practice had a system in place to identify patients with a fragility fracture for the purpose of fracture risk assessments, but only 87 (28.1%) indicated that these patients were actively invited for assessment. Similarly, 108 respondents (34.8%) noted the presence of a system to identify patients on systemic glucocorticoids, while only 69 (22.3%) reported routinely inviting these patients for assessment. For patients with a history of falls, 72 respondents (23.2%) acknowledged a system for identification, yet just 64 (20.7%) indicated that these patients were invited for fracture risk assessment. Notably, more than one-third of respondents selected “do not know” when asked about the existence of systems for case identification and routine invitations for assessment (Supplementary Data 6).

The majority of responding HCPs and non-HCPs (212, 68.4%) reported that their practice invited all patients prescribed osteoporosis medications for a medication review. Additionally, 138 respondents (44.5%) indicated that their practice had a system in place by which they could identify long-term bisphosphonate users for the purposes of treatment reviews (Supplementary Data 6).

### Challenges in osteoporosis care

Approximately three quarters of responding HCPs considered their knowledge of osteoporosis care to be adequate for providing effective, quality care for patients (217, 74.8%). More HCPs rated their knowledge to be low (55, 19.0%) than high (17, 5.9%).

HCPs were asked to select, from a series of statements, strategies that could improve the care they provide to individuals at risk osteoporotic fracture. The 291 respondents rated “improved education about osteoporosis during postgraduate training” (182, 62.5%) and “tools to aid communication with patients about osteoporosis medicines” (*n* = 177, 60.8%) most highly (Table 3).

**Table 3:** Strategies to improve the care delivered to people with, or at risk of, osteoporosis

Strategies	n (%)*
Improved education about osteoporosis during postgraduate training	182 (62.54)
Tools to aid communication with patients around the risk/benefit of osteoporosis medicines	177 (60.82)
More time to see patients	175 (60.14)
Accredited training course for primary care practitioners	152 (52.23)
More primary care staff	148 (50.86)
Upskilling of other members of the primary care workforce	143 (49.14)
Inclusion of treatment recommendations with bone density scan reports	131 (45.02)
improved education about osteoporosis during undergraduate training	122 (41.92)
Increased access to specialist (secondary care) advice	115 (39.52)
Spending time in, or observing, secondary care led metabolic bone disease clinics	111 (38.14)
Clearer secondary care referral criteria	107 (36.77)
Clearer national guidelines	105 (36.08)
Increased access to bone density scans	61 (20.96)
Other	8 (2.75)

*n* = 291, HCPs

*Participants were not limited to the number of responses and so totals are greater than the number of respondents

### Free text responses

In total, 86 respondents provided free text response to the question: “Are there any other comments or suggestions you would like to make about the care of people with, or at risk of, osteoporosis in primary care settings?” Reflexive thematic analysis identified four overarching themes: Challenging Decision-Making Processes; Discontent with Secondary Care Responsibilities; Insufficient prioritisation, incentivisation and remuneration; Inadequate Access to Resources. Themes and subthemes with exemplar quotes are outlined in Table 4.

**Table 4:** Overview of the reflexive thematic analysis of free text comments

Theme	Example codes	Example free text comment(s)
**Challenging Decision-Making Processes** *This theme highlights the multifaceted challenges HCPs face when making decisions about osteoporosis care. These challenges stem from limited knowledge, suboptimal clinical skills and inadequate clinical guidance*	Poor knowledge and understanding of osteoporosis	“This has highlighted my appalling lack of knowledge” ID 753, Advanced Nurse Practitioners
	Suboptimal clinical skills	“I find the knowledge of my colleagues (GPs) is limited to prescribing in line with secondary care recommendation or sending for DXAs, there seems to be a lack of confidence around prescribing based on FRAX alone where indicated, and a lack of knowledge on alternative treatment options. There is also a lack of confidence around drug holidays/treatment breaks without DEXA, which has impacted patient care with DEXA delays due to COVID.” ID 487, Pharmacist
	Inadequate clinical guidance	“Have to say, I do not like osteoporosis. Guidelines seem to change often. Don’t consult with enough patients to know off by heart so always have to refer to guidelines, which is tedious and time-consuming. Helpful when DXA reports tell you what to do! But this isn’t consistent.” ID 933, GP
**Discontent with Secondary Care Responsibilities** *This theme reflects broader systemic issues, including unclear delineation of responsibilities, inefficiencies in communication between primary and secondary care, and tensions around the perceived value and role of FLS*	Role of secondary care services	“Secondary care take no ownership of this as an issue or problem it is a source of unnecessary transfer of work to primary care all too frequently. We frequently see people who have been admitted to hospital with a fracture, seen in fracture clinic or the general medical wards and the discharge letter will ask the GP to refer to a bone health clinic or consider prescribing or pass no comment about future fracture risk. This should be being addressed during inpatient care. I think the orthopaedic doctors should take responsibility and ownership of this.” ID 725, GP
**Insufficient prioritisation, incentivisation and remuneration** *This theme underscores the tension between HCPs’ professional dedication to improving osteoporosis care and the structural barriers, including time constraints and lack of financial incentivisation, that hinder their ability to deliver optimal care*	Prioritisation	“It is a silent subject and not glamorous and does not get news headlines. If there was a news story that said 20 000 older people have fallen and fractured a hip this year and 4000 of them died it would start a debate.” ID 807, GP
	Incentivisation (Quality and Outcomes Framework)	“Quality and Outcomes Framework indicators need to expand again to include the recommendations suggested to gain any increased attention, otherwise, it is essentially unfunded work.” ID 176, GP
	Lack of time	“I believe we could improve patient care by looking systemically, however, this takes extra TIME in an already stretched service.” ID 1144, GP
**Inadequate Access to Resources** *This theme captures the systemic constraints that hinder proactive osteoporosis care in primary care settings. HCPs and non-HCPs reported barriers related to access to essential clinical services, including bone density scans, dental care and appropriately resourced clinical systems*	Limited access to bone density scans	“Since changes to treatments intervals and post COVID limitation to DXA scans I am baffled. I dread the review bone density tasks set popping up. Half the time I get a DXA and the other half the scan is rejected but without explanation. All GPs in my practice are confused too.” ID 1445, GP
	Limited access to dental services	“When counselling patients on bisphosphates I often find the lack of access to dentists for check-ups problematic. Ideally I would want to ensure patients are up to date and have regular reviews, but more often than not this is not possible.” ID 642, GP
	Inadequate clinical systems	“The EMIS templates don't have an inbuilt FRAX calculator—this needs to be built into EMIS and not accessed via a weblink to an online tool—it takes too long to calculate in this way, so many GPs don’t do the scores.” ID 725, GP

Primary care HCPs described discontent with currently perceived secondary care responsibilities and noted gaps in interprofessional collaboration that undermined optimal osteoporosis care delivery. Particularly, FLSs were criticised for not starting medication or leading to deskilling of primary care staff. Challenging decision-making processes were influenced by limited knowledge, suboptimal clinical skills and inadequate clinical guidance, which was described as confusing and conflicting, leaving many HCPs uncertain about managing complex cases, interpreting investigations and implementing management strategies. These issues appear to be compounded by inadequate access to resources, including restricted and delayed access to bone density scans, insufficient dental care and clinical systems lacking integrated tools like fracture risk calculators or automated recalls. Finally, time pressures and competing priorities constrained HCPs capacity to provide comprehensive care, as competing clinical demands and high workloads appear to relegate osteoporosis care to a lower priority. The current osteoporosis Quality and Outcomes Framework, which provides financial incentives to general practices based on their performance on defined quality indicators in England, Wales and Northern Ireland, was deemed to be inadequate for prioritising osteoporosis care.

## Discussion

This study represents the first national e-survey of UK primary care staff, including the role of non-medical HCPs, in routine osteoporosis primary care. The findings demonstrate that while respondents identified osteoporosis as an important condition, many gaps in confidence with specific osteoporosis skills were reported, in addition to a general lack of systematic approaches to assessment and follow-up of people with the condition. Fracture risk assessments appear to be conducted opportunistically rather than systematically, particularly in people with a history of falls. Free text findings suggest that this is influenced by competing priorities and inadequate resources including DXA and dentistry services.

In contrast to other research which reports that primary care physicians do not consider osteoporosis as a priority [36–40], respondents in this survey felt osteoporosis was important, but reported other barriers to care. Other attitudinal findings of interest are the relatively high levels of concern among both respondents about the safety of osteoporosis medication, which has been reported as a contributing factor to the osteoporosis “treatment gap” [5].

Lack of clarity over professional roles, particularly between primary and secondary care, has been identified in our groups previous research which demonstrated that GPs are unclear as to whether FLSs have counselled patients on medication [41], and may perceive secondary care should take a greater role in the identification and management of people with fractures or secondary causes of osteoporosis [24, 42]. These findings add further to this evidence by demonstrating a large degree of uncertainty that prevails over the existence of services (e.g. local FLSs) or nature of other roles. The free text findings also demonstrate the complexity of the clinical decision-making in osteoporosis and that existing clinical guidelines are perhaps not effective at supporting this decision-making, given that limited awareness of these was reported, and they were reported as conflicting and confusing. Other systemic barriers to care identified in free text responses include the lack of appropriate remuneration and resources, difficulty with integration of IT systems and lack of automated or systematic approaches to identification of people with risk factors.

The novel inclusion in this survey of non-HCPs was driven by public contributors who told us that the views of non-HCPs are valuable as non-clinical staff (e.g. receptionists) can be important gatekeepers to accessing primary care. Responding non-HCPs generally did not feel that they had a good understanding of the condition, and were also less aware that drugs could lower fracture risk, perhaps indicative of general lack of societal awareness of the condition and its treatment. However, given the small number of non-HCP respondents, this is an area that requires further research.

Results suggest that information about osteoporosis medicines is not routinely provided to patients and that dedicated medication reviews are uncommon, with osteoporosis medicine most often being reviewed in consultations covering all the medicines a patient is taking. Given that persistence with oral bisphosphonates at 2 years is between 12.9% and 60.6%, and poor persistence reduces medication efficacy [43, 44], structured osteoporosis medication reviews could help address this issue. Patient-reported reasons for discontinuing osteoporosis medicines include unmet information needs and lack of systematic follow-up [45], suggesting that improved review processes and provision of adequate information may support long-term persistence.

### Implications for practice and research

Previous surveys and qualitative studies have reported that primary care HCPs report low levels of confidence, knowledge and skills in osteoporosis care [10, 13, 36, 37]. However, this survey adds to this literature by demonstrating potential opportunities to address these issues. For example, practice-based pharmacists appear confident in the delivery of osteoporosis care, particularly with reference to medication management. The role of practice-based pharmacists in UK primary care is rapidly expanding and evidence suggests improved patient outcomes in other chronic conditions [46, 47]. It is therefore the authors opinion that practice-based pharmacists may be well placed, with appropriate support, to take on many aspects of osteoporosis care including assessment, initiating and following up people receiving pharmacological treatment. However, more work is needed in this area.

Collectively, the interconnected barriers identified in free text comments reflect a systemic need for clearer role definitions, enhanced incentivisation strategies and better-designed clinical systems to support evidence-based osteoporosis management in primary care. With regard to incentivisation, research is needed to identify the measurable quality indicators that will lead to cost savings and tangible patient benefit. Furthermore, automated approaches are needed, and have already shown promise, in facilitating systematic identification of those at increased risk, and in need of follow-up [48]. There is also a need to ensure that clinical guidelines are readily accessible to, and meet the needs of primary care HCPs, with a clearly described follow-up pathway for people receiving osteoporosis medication.

The findings of this e-survey have informed a subsequent qualitative interview study designed to further explore and explain the observed results. Together, these findings will inform practical strategies and interventions to enhance the identification, management, and treatment of patients at risk of osteoporosis in primary care settings.

### Strengths and limitations

A major strength of the e-survey is the high level of participant recruitment, which exceeded target and was higher than previous UK surveys of primary care staff focused on other musculoskeletal conditions [25]. Response was also comparable to, or exceeded, international primary care surveys on osteoporosis [10, 13, 15].

This e-survey is subject to limitations. Public contributors, via both the osteoporosis Research User Group and the Community of Practice, strengthened the design, content and interpretation of results, ensuring alignment with patient priorities. However, challenges were encountered in achieving public representation from underserved groups and we recommend that future studies specifically allocate resources to improving engagement (e.g. collaboration with community leaders).

The UK-specific healthcare context may limit generalisability to other primary care systems, where the roles of non-GP HCPs, access to osteoporosis investigations and guideline adoption may differ. Potential sources of bias include self-selection, with respondents possibly having a greater interest in osteoporosis. Missing data was another limitation, as e-survey questions were not mandatory to encourage participation. While the e-survey was informed by existing literature, previous surveys and expert opinion, the absence of validated measures may limit comparability with other studies. Finally, NHS workforce data suggests that respondents may underrepresent non-white heritage groups, given that 49.9% of doctors working in hospital or community settings are identify as from minoritised ethnic groups [49].

## Conclusion

This e-survey highlights that while primary care HCPs recognise the importance of osteoporosis care, challenges remain in narrowing the treatment gap. Barriers include a lack of confidence in key clinical skills such as interpreting bone density scans, performing fracture risk assessments and making long-term treatment decisions. Additionally, gaps in evidence-based care are evident, with a lack of systematic case finding in accordance with national guidance and limited osteoporosis medication reviews. These challenges are further compounded by system-level constraints such as insufficient time and delays in DXA access.

Despite these barriers, the survey identifies potential enablers for improving care. HCPs expressed a strong desire for enhanced postgraduate education and tools to support risk/benefit communication. Free text comments further emphasise the need for revised remuneration models and improved IT systems, including automated case finding. Additionally, the evolving role of non-GP HCPs, such as Pharmacists, in osteoporosis care warrants further exploration.

## Supplementary Information

Below is the link to the electronic supplementary material.

ESM 1 (DOCX 118 KB)

## References

[1] Willers C, Norton N, Harvey NC et al (2022) Osteoporosis in Europe: a compendium of country-specific reports. Arch Osteoporos 17:1–129. 10.1007/S11657-021-00969-8/METRICS 10.1007/s11657-021-00969-8 PMC8789736 35079919

[2] Borgström F, Karlsson L, Ortsäter G et al (2020) Fragility fractures in Europe: burden, management and opportunities. Arch Osteoporos 15:59. 10.1007/S11657-020-0706-Y 32306163 10.1007/s11657-020-0706-y PMC7166207

[3] Kanis JA, Cooper C, Rizzoli R et al (2019) European guidance for the diagnosis and management of osteoporosis in postmenopausal women. Osteoporos Int 30:3. 10.1007/S00198-018-4704-5 30324412 10.1007/s00198-018-4704-5 PMC7026233

[4] Eastell R, Rosen CJ, Black DM et al (2019) Pharmacological management of osteoporosis in postmenopausal women: an endocrine society* clinical practice guideline. J Clin Endocrinol Metab 104:1595–1622. 10.1210/jc.2019-00221 30907953 10.1210/jc.2019-00221

[5] Fuggle NR, Curtis B, Clynes M et al (2021) The treatment gap: the missed opportunities for osteoporosis therapy. Bone 144:115833. 10.1016/J.BONE.2020.115833 33359889 10.1016/j.bone.2020.115833 PMC7116600

[6] Kanis JA, Svedbom A, Harvey N, McCloskey EV (2014) The osteoporosis treatment gap. J Bone Miner Res 29:1926–1928. 10.1002/JBMR.2301 24956507 10.1002/jbmr.2301

[7] McCloskey E, Rathi J, Heijmans S et al (2021) The osteoporosis treatment gap in patients at risk of fracture in European primary care: a multi-country cross-sectional observational study. Osteoporos Int 32:251–259. 10.1007/S00198-020-05557-Z/TABLES/4 32829471 10.1007/s00198-020-05557-z PMC7838133

[8] Royal Osteoporosis Society (2021) APPG on osteoporosis and bone health. Inquiry Report: How to end the postcode lottery for access to a quality fracture liaison service. https://strwebprdmedia.blob.core.windows.net/media/31tbj2dt/appg-on-osteoporosis-and-bone-health-fls-inquiry-inquiry-report-2021.pdf

[9] Bullock L, Abdelmagid S, Fleming J et al (2023) Variation in UK fracture liaison service consultation conduct and content before and during the COVID pandemic: results from the iFraP-D UK survey. Arch Osteoporos 19:5. 10.1007/s11657-023-01361-4 38123745 10.1007/s11657-023-01361-4 PMC10733195

[10] Choong DS, Tan NC, Koh YLE et al (2023) Osteoporosis management by primary care physicians in Singapore: a survey on osteoporosis guidelines utilisation and barriers to care. Arch Osteoporos 18:72. 10.1007/s11657-023-01283-1 37209254 10.1007/s11657-023-01283-1 PMC10198784

[11] Gupta ED, Goh EML, Gun SC et al (2013) Osteoporosis awareness among primary care physicians in Malaysia. EXCLI J 12:521–522 27034635 PMC4803010

[12] Chenot R, Scheidt-Nave C, Gabler S et al (2007) German primary care doctors’ awareness of osteoporosis and knowledge of national guidelines. Exp Clin Endocrinol Diabetes 115:584–589. 10.1055/S-2007-981454/ID/20 17943692 10.1055/s-2007-981454

[13] Tay CL, Ng WL, Beh HC et al (2022) Screening and management of osteoporosis: a survey of knowledge, attitude and practice among primary care physicians in Malaysia. Arch Osteoporos 17:72. 10.1007/s11657-022-01111-y 35474021 10.1007/s11657-022-01111-y PMC9041673

[14] Pérez-Edo L, Ciria Recasens M, Castelo-Branco C et al (2004) Management of osteoporosis in general practice: a cross-sectional survey of primary care practitioners in Spain. Osteoporos Int 15:252–257. 10.1007/s00198-003-1569-y 14745487 10.1007/s00198-003-1569-y

[15] Fogelman Y, Goldshtein I, Segal E, Ish-Shalom S (2016) Managing osteoporosis: a survey of knowledge, attitudes and practices among primary care physicians in Israel. PLoS ONE 11:e0160661. 10.1371/JOURNAL.PONE.0160661 27494284 10.1371/journal.pone.0160661 PMC4975485

[16] Alawi ZS, Matar E, Hasan WF et al (2025) Osteoporosis in primary care: an analysis of family physicians’ knowledge, attitudes, and practices in Bahrain. Cureus. 10.7759/cureus.79968 40182354 10.7759/cureus.79968 PMC11966336

[17] Taylor JC, Sterkel B, Utley M et al (2001) Opinions and experiences in general practice on osteoporosis prevention, diagnosis and management. Osteoporos Int 12:844–848. 10.1007/S001980170035/METRICS 11716187 10.1007/s001980170035

[18] NHS England (2023) NHS long term workforce plan. https://www.england.nhs.uk/long-read/nhs-long-term-workforce-plan-2/

[19] NICE (2017) Osteoporosis: assessing the risk of fragility fracture. Clinical guideline [CG146]. https://www.nice.org.uk/guidance/cg146

[20] Gregson CL, Armstrong DJ, Bowden J et al (2022) UK clinical guideline for the prevention and treatment of osteoporosis. Archives of Osteoporosis 17:1–46. 10.1007/S11657-022-01061-5 10.1007/s11657-022-01061-5 PMC8979902 35378630

[21] NICE (2017) Bisphosphonates for treating osteoporosis. Technology appraisal guidance [TA464]. https://www.nice.org.uk/guidance/ta464

[22] NICE (2017) Osteoporosis quality standard [QS149]. https://www.nice.org.uk/guidance/qs149

[23] SIGN (2021) Management of osteoporosis and the prevention of fragility fractures: a national clinical guideline. SIGN (SIGN publication no. 142). https://www.sign.ac.uk/media/1812/sign-142-osteoporosis-v3.pdf

[24] Hawarden A, Boylan J, Cox N et al (2023) Primary care practitioners’ views on barriers and enablers of osteoporosis care: a scoping review. JBMR Plus 7:e10815. 10.1002/jbm4.10815

[25] Kingsbury SR, Conaghan PG (2012) Current osteoarthritis treatment, prescribing influences and barriers to implementation in primary care. Prim Health Care Res Dev 13:373–381. 10.1017/S1463423612000072 22464219 10.1017/S1463423612000072

[26] Broadbent E, Petrie KJ, Main J, Weinman J (2006) The Brief Illness Perception Questionnaire. J Psychosom Res 60:631–637. 10.1016/J.JPSYCHORES.2005.10.020 16731240 10.1016/j.jpsychores.2005.10.020

[27] Horne R, Weinman J, Hankins M (1999) The beliefs about medicines questionnaire: the development and evaluation of a new method for assessing the cognitive representation of medication. Psychol Health 14:1–24. 10.1080/08870449908407311

[28] Office for National Statistics (2022) Ethnic group, England and Wales: Census 2021. https://www.ons.gov.uk/peoplepopulationandcommunity/culturalidentity/ethnicity/bulletins/ethnicgroupenglandandwales/census2021. Accessed Jan 2025

[29] Staniszewska S, Brett J, Simera I et al (2017) GRIPP2 reporting checklists: tools to improve reporting of patient and public involvement in research. BMJ (Clinical research ed) 358:j3453. 10.1136/BMJ.J3453 10.1136/bmj.j3453 PMC5539518 28768629

[30] Edwards P, Roberts I, Clarke M et al (2023) Methods to increase response to postal and electronic questionnaires. Cochrane Database Syst Rev. 10.1002/14651858.MR000008.pub5 38032037 10.1002/14651858.MR000008.pub5 PMC10687884

[31] Connelly LM (2016) Understanding research. Fisher’s Exact Test MEDSURG Nursing 25:58–61 27044131

[32] Braun V, Clarke V, Boulton E et al (2021) The online survey as a qualitative research tool. Int J Soc Res Methodol 24:641–654. 10.1080/13645579.2020.1805550

[33] Braun V, Clarke V (2022) Thematic analysis: A practical guide to understanding and doing. London: SAGE publications

[34] Braun V, Clarke V (2019) Reflecting on reflexive thematic analysis. Qual Res Sport, Exerc Health 11:589–597. 10.1080/2159676X.2019.1628806

[35] Royal Osteoporosis Society (2017) Quality standards for osteoporosis and prevention of fragility fractures. https://theros.org.uk/media/0dillsrh/ros-op-standards-november-2017.pdf

[36] Otmar R, Reventlow SD, Nicholson GC et al (2012) General medical practitioners’ knowledge and beliefs about osteoporosis and its investigation and management. Arch Osteoporos 7:107–114. 10.1007/S11657-012-0088-X 23225288 10.1007/s11657-012-0088-x

[37] Salminen H, Piispanen P, Toth-Pal E (2019) Primary care physicians’ views on osteoporosis management: a qualitative study. Arch Osteoporos 14:48. 10.1007/S11657-019-0599-9 31028556 10.1007/s11657-019-0599-9 PMC6486622

[38] Bishop S, Narayanasamy MJ, Paskins Z et al (2023) Clinicians’ views of prescribing oral and intravenous bisphosphonates for osteoporosis: a qualitative study. BMC Musculoskelet Disord 24:1–10. 10.1186/S12891-023-06865-1/TABLES/1 10.1186/s12891-023-06865-1 PMC10540377 37770860

[39] Merle B, Haesebaert J, Bedouet A et al (2019) Osteoporosis prevention: where are the barriers to improvement in French general practitioners? A qualitative study. PloS one 14:e0219681. 10.1371/JOURNAL.PONE.0219681 10.1371/journal.pone.0219681 PMC6634405 31310619

[40] Bennett MJ, Center JR, Perry L (2023) Exploring barriers and opportunities to improve osteoporosis care across the acute-to-primary care interface: a qualitative study. Osteoporos Int 34:1249–1262. 10.1007/S00198-023-06748-0/TABLES/3 37093239 10.1007/s00198-023-06748-0 PMC10281902

[41] Bullock L, Manning F, Hawarden A et al (2024) Exploring practice and perspectives on shared decision-making about osteoporosis medicines in Fracture Liaison Services: the iFraP development qualitative study. Arch Osteoporos 19:50. 10.1007/s11657-024-01410-6 38898212 10.1007/s11657-024-01410-6 PMC11186902

[42] Hawarden A, Bullock L, Chew-Graham CA et al (2023) Incorporating FRAX into a nurse-delivered integrated care review: a multi-method qualitative study. BJGP Open 7:BJGPO.2022.0146. 10.3399/BJGPO.2022.0146 10.3399/BJGPO.2022.0146 PMC10354387 36746471

[43] Imaz I, Zegarra P, González-Enríquez J et al (2010) Poor bisphosphonate adherence for treatment of osteoporosis increases fracture risk: systematic review and meta-analysis. Osteoporos Int 21:1943–1951. 10.1007/S00198-009-1134-4/FIGURES/7 19967338 10.1007/s00198-009-1134-4

[44] Fatoye F, Smith P, Gebrye T, Yeowell G (2019) Real-world persistence and adherence with oral bisphosphonates for osteoporosis: a systematic review. BMJ open 9:e027049. 10.1136/BMJOPEN-2018-027049 30987990 10.1136/bmjopen-2018-027049 PMC6500256

[45] Hawarden A, Jinks C, Mahmood W et al (2020) Public priorities for osteoporosis and fracture research: results from a focus group study. Arch Osteoporos 15:1–10. 10.1007/S11657-020-00766-9/TABLES/2 10.1007/s11657-020-00766-9 PMC7297850 32548718

[46] Claire M, Claire A, Matthew B (2022) The role of clinical pharmacists in general practice in England: Impact, perspectives, barriers and facilitators. Res Social Adm Pharm 18:3432–3437. 10.1016/j.sapharm.2021.10.006 34802958 10.1016/j.sapharm.2021.10.006

[47] Anderson M, Francetic I (2025) Adoption of clinical pharmacist roles in primary care: longitudinal evidence from English general practice. Br J Gen Pract 75:e173–e180. 10.3399/BJGP.2024.0320 39317390 10.3399/BJGP.2024.0320 PMC11800411

[48] Nedungayil SK, McCloskey EV, Davis S (2024) P1184 Implementing improved treatment for patients at high risk of fracture in primary care: experiences from a hybrid digital and clinical evaluation model of care. Aging Clin Exp Res 36:174. 10.1007/s40520-024-02766-y

[49] NHS England (2022) NHS workforce statistics - June 2022 (including selected provisional statistics for July 2022). https://digital.nhs.uk/data-and-information/publications/statistical/nhs-workforce-statistics/june-2022. Accessed Jan 2025

