# Supplementary material for: Osteoporosis care in primary care settings: a national UK e-survey
Source: Arch Osteoporos. 2025 Aug 6;20(1):109. doi: 10.1007/s11657-025-01591-8 (PMC12325544; doi:10.1007/s11657-025-01591-8)
Supplement: Supplementary file 1 — (DOCX 118 KB) [file 11657_2025_1591_MOESM1_ESM.docx]

# Supplementary Material

## Supplementary Data 1 – Full E-Survey

See PDF.

## Supplementary Data 2 – Beliefs About Osteoporosis

### HCPs

| Statement (n responders) | Strongly agree | Agree | Neither agree or disagree | Disagree | Strongly disagree |
| --- | --- | --- | --- | --- | --- |
|  | n (%) | n (%) | n (%) | n (%) | n (%) |
| I think osteoporosis is an important condition (309) | 142 (45.95) | 157 (50.81) | 5 (1.62) | 0 (0.00) | 5 (1.62) |
| People's lives are affected by osteoporosis and osteoporotic fractures (309) | 229 (74.11) | 78 (25.24) | 1 (0.32) | 0 (0.0) | 1 (0.32) |
| I have a good understanding of osteoporosis (308) | 38 (12.34) | 185 (60.06) | 61 (19.81) | 23 (7.47) | 1 (0.32) |
| Osteoporosis is an inevitable consequence of ageing (307) | 8 (2.61) | 43 (14.01) | 81 (26.38) | 162 (52.77) | 13 (4.23) |
| Lifestyle interventions (e.g. weight bearing exercise or smoking cessation) are effective in reducing the risk of fracture (309) | 138 (44.66) | 168 (54.37) | 3 (0.97) | 0 (0.00) | 0 (0.00) |
| Osteoporosis medicines are effective in reducing the risk of fracture (307) | 95 (30.94) | 184 (59.93) | 21 (6.84) | 5 (1.63) | 2 (0.65) |
| I sometimes worry about osteoporosis medicines causing unpleasant side effects (308) | 40 (12.99) | 188 (61.04) | 61 (19.81) | 18 (5.84) | 1 (0.32) |
| I sometimes worry about the long-term side effects of osteoporosis medicines (e.g. osteonecrosis of the jaw or atypical femoral fracture) (307) | 30 (9.77) | 174 (56.68) | 73 (23.78) | 25 (8.14) | 5 ( 1.63) |

### Non-HCPs

| Statement (n responders) | Strongly agree | Agree | Neither agree or disagree | Disagree | Strongly disagree |
| --- | --- | --- | --- | --- | --- |
|  | n (%) | n (%) | n (%) | n (%) | n (%) |
| I think osteoporosis is an important condition (29) | 12 (41.38) | 14 (48.28) | 2 (6.90) | 0 (0.00) | 1 (3.45) |
| People's lives are affected by osteoporosis and osteoporotic fractures (29) | 14 (48.28) | 14 (48.28) | 0 (0.00) | 0 (0.00) | 1 (3.45) |
| I have a good understanding of osteoporosis (29) | 0 (0.00) | 5 (17.24) | 14 (48.28) | 8 (27.59) | 2 (6.90) |
| Osteoporosis is an inevitable consequence of ageing (29) | 0 (0.00) | 5 (17.24) | 14 (48.28) | 8 (27.59) | 2 (6.90) |
| Lifestyle interventions (e.g. weight bearing exercise or smoking cessation) are effective in reducing the risk of fracture (27) | 5 (18.52) | 15 (55.56) | 6 (22.22) | 0 (0.00) | 1 (3.70) |
| Osteoporosis medicines are effective in reducing the risk of fracture (25) | 4 (16.00) | 13 (52.00) | 7 (28.00) | 0 (0.00) | 1 (4.00) |

### Role Comparison of HCP Beliefs About Osteoporosis (GP, Practice Nurse, Advanced Nurse Practitioner and Pharmacist)

| **I think osteoporosis is an important condition** | | | |
| --- | --- | --- | --- |
| **Professional Role** | **Agree** | **Do not agree** |  |
|  | **n (%)** | **n (%)** | **p-value** |
| General Practitioner | 195 (98.48) | 3 (1.52) | 0.123 |
| Practice Nurse | 27 (93.10) | 2 (6.90) |  |
| General Practitioner | 195 (98.48) | 3 (1.52) | 0.403 |
| Advanced Nurse Practitioner | 26 (96.30) | 1 (3.70) |  |
| General Practitioner | 195 (98.48) | 3 (1.52) | 0.255 |
| Pharmacist | 14 (93.33) | 1 (6.67) |  |
| Practice Nurse | 27 (93.10) | 2 (6.90) | 1 |
| Advanced Nurse Practitioner | 26 (96.30) | 1 (3.70) |  |
| Practice Nurse | 27 (93.10) | 2 (6.90) | 1 |
| Pharmacist | 14 (93.33) | 1 (6.67) |  |
| Advanced Nurse Practitioner | 26 (96.30) | 1 (3.70) | 1 |
| Pharmacist | 14 (93.33) | 1 (6.67) |  |

| **I have a good understanding of osteoporosis** | | | |
| --- | --- | --- | --- |
| **Professional Role** | **Agree** | **Do not agree** |  |
|  | **n (%)** | **n (%)** | **p-value** |
| General Practitioner | 161 (81.31) | 37 (18.69) | <0.001* |
| Practice Nurse | 12 (42.86) | 16 (57.14) |  |
| General Practitioner | 161 (81.31) | 37 (18.69) | <0.001* |
| Advanced Nurse Practitioner | 13 (48.15) | 14 (51.85) |  |
| General Practitioner | 161 (81.31) | 37 (18.69) | 0.496 |
| Pharmacist | 11 (73.33) | 4 (26.67) |  |
| Practice Nurse | 12 (42.86) | 16 (57.14) | 0.789 |
| Advanced Nurse Practitioner | 13 (48.15) | 14 (51.85) |  |
| Practice Nurse | 12 (42.86) | 16 (57.14) | 0.107 |
| Pharmacist | 11 (73.33) | 4 (26.67) |  |
| Advanced Nurse Practitioner | 13 (48.15) | 14 (51.85) | 0.193 |
| Pharmacist | 11 (73.33) | 4 (26.67) |  |

| **Osteoporosis is an inevitable consequence of ageing** | | | |
| --- | --- | --- | --- |
| **Professional Role** | **Agree** | **Do not agree** |  |
|  | **n (%)** | **n (%)** | **p-value** |
| General Practitioner | 40 (20.41) | 156 (79.59) | 0.036* |
| Practice Nurse | 1 (3.45) | 28 (96.55) |  |
| General Practitioner | 40 (20.41) | 156 (79.59) | 0.034* |
| Advanced Nurse Practitioner | 1 (3.70) | 26 (96.30) |  |
| General Practitioner | 40 (20.41) | 156 (79.59) | 0.741 |
| Pharmacist | 2 (14.29) | 12 (85.71) |  |
| Practice Nurse | 1 (3.45) | 28 (96.55) | 1 |
| Advanced Nurse Practitioner | 1 (3.70) | 26 (96.30) |  |
| Practice Nurse | 1 (3.45) | 28 (96.55) | 0.243 |
| Pharmacist | 2 (14.29) | 12 (85.71) |  |
| Advanced Nurse Practitioner | 1 (3.70) | 26 (96.30) | 0.265 |
| Pharmacist | 2 (14.29) | 12 (85.71) |  |

| **Lifestyle interventions (e.g. weight bearing exercise or smoking cessation) are effective in reducing the risk of fracture** | | | |
| --- | --- | --- | --- |
| **Professional Role** | **Agree** | **Do not agree** |  |
|  | **n (%)** | **n (%)** | **p-value** |
| General Practitioner | 197 (99.49) | 1 (0.51) | 1 |
| Practice Nurse | 29 (100.00) | 0 (0.00) |  |
| General Practitioner | 197 (99.49) | 1 (0.51) | 0.226 |
| Advanced Nurse Practitioner | 26 (96.30) | 1 (3.70) |  |
| General Practitioner | 197 (99.49) | 1 (0.51) | 1 |
| Pharmacist | 15 (100.00) | 0 (0.00) |  |
| Practice Nurse | 29 (100.00) | 0 (0.00) | 0.482 |
| Advanced Nurse Practitioner | 26 (96.30) | 1 (3.70) |  |
| Practice Nurse | 29 (100.00) | 0 (0.00) | N/A |
| Pharmacist | 15 (100.00) | 0 (0.00) |  |
| Advanced Nurse Practitioner | 26 (96.30) | 1 (3.70) | 1 |
| Pharmacist | 15 (100.00) | 0 (0.00) |  |

| **Osteoporosis medicines are effective in reducing the risk of fracture** | | | |
| --- | --- | --- | --- |
| **Professional Role** | **Agree** | **Do not agree** |  |
|  | **n (%)** | **n (%)** | **p-value** |
| General Practitioner | 183 (93.37) | 13 (6.63) | 0.007* |
| Practice Nurse | 22 (75.86) | 7 (24.14) |  |
| General Practitioner | 183 (93.37) | 13 (6.63) | 0.7 |
| Advanced Nurse Practitioner | 25 (92.59) | 2 (7.41) |  |
| General Practitioner | 183 (93.37) | 13 (6.63) | 0.605 |
| Pharmacist | 15 (100.00) | 0 (0.00) |  |
| Practice Nurse | 22 (75.86) | 7 (24.14) | 0.146 |
| Advanced Nurse Practitioner | 25 (92.59) | 2 (7.41) |  |
| Practice Nurse | 22 (75.86) | 7 (24.14) | 0.077 |
| Pharmacist | 15 (100.00) | 0 (0.00) |  |
| Advanced Nurse Practitioner | 25 (92.59) | 2 (7.41) | 0.53 |
| Pharmacist | 15 (100.00) | 0 (0.00) |  |

| **I sometimes worry about osteoporosis medicines causing unpleasant side effects** | | | |
| --- | --- | --- | --- |
| **Professional Role** | **Agree** | **Do not agree** |  |
|  | **n (%)** | **n (%)** | **p-value** |
| General Practitioner | 160 (81.22) | 37 (18.78) | 0.001* |
| Practice Nurse | 15 (51.72) | 14 (48.28) |  |
| General Practitioner | 160 (81.22) | 37 (18.78) | 0.203 |
| Advanced Nurse Practitioner | 19 (70.37) | 8 (29.63) |  |
| General Practitioner | 160 (81.22) | 37 (18.78) | 0.184 |
| Pharmacist | 10 (66.67) | 5 (33.33) |  |
| Practice Nurse | 15 (51.72) | 14 (48.28) | 0.18 |
| Advanced Nurse Practitioner | 19 (70.37) | 8 (29.63) |  |
| Practice Nurse | 15 (51.72) | 14 (48.28) | 0.522 |
| Pharmacist | 10 (66.67) | 5 (33.33) |  |
| Advanced Nurse Practitioner | 19 (70.37) | 8 (29.63) | 1 |
| Pharmacist | 10 (66.67) | 5 (33.33) |  |

| **I sometimes worry about the long-term side effects of osteoporosis medicines (e.g. osteonecrosis of the jaw or atypical femoral fracture)** | | | |
| --- | --- | --- | --- |
| **Professional Role** | **Agree** | **Do not agree** |  |
|  | **n (%)** | **n (%)** | **p-value** |
| General Practitioner | 144 (73.10) | 53 (26.90) | 0.001* |
| Practice Nurse | 11 (39.29) | 17 (60.71) |  |
| General Practitioner | 144 (73.10) | 53 (26.90) | 0.071 |
| Advanced Nurse Practitioner | 15 (55.56) | 12 (44.44) |  |
| General Practitioner | 144 (73.10) | 53 (26.90) | 1 |
| Pharmacist | 11 (73.33) | 4 (26.67) |  |
| Practice Nurse | 11 (39.29) | 17 (60.71) | 0.285 |
| Advanced Nurse Practitioner | 15 (55.56) | 12 (44.44) |  |
| Practice Nurse | 11 (39.29) | 17 (60.71) | 0.055 |
| Pharmacist | 11 (73.33) | 4 (26.67) |  |
| Advanced Nurse Practitioner | 15 (55.56) | 12 (44.44) | 0.33 |
| Pharmacist | 11 (73.33) | 4 (26.67) |  |

## Supplementary Data 3 – HCP Confidence in Delivering Osteoporosis Care

| Statement (n responders) | Strongly agree | Agree | Neither agree or disagree | Disagree | Strongly disagree |
| --- | --- | --- | --- | --- | --- |
|  | n (%) | n (%) | n (%) | n (%) | n (%) |
| I am confident that I can explain osteoporosis in a way that patients understand (305) | 70 (22.95) | 181 (59.34) | 31 (10.16) | 22 (7.21) | 1 (0.33) |
| I am confident that I can identify patients who require a fracture risk assessment (304) | 47 (15.46) | 151 (49.67) | 52 (17.11) | 52 (17.11) | 2 (0.66) |
| I am confident that I can perform a fracture risk assessment (304) | 64 (21.05) | 140 (46.05) | 43 (14.14) | 50 (16.45) | 7 (2.30) |
| I am confident that I can explain fracture risk assessment results in a way that patients understand (304) | 41 (13.49) | 148 (48.68) | 59 (19.41) | 52 (17.11) | 4 (1.32) |
| I am confident interpreting the numeric results of bone density scans (for example, interpreting T-scores) (302) | 38 (12.58) | 106 (35.10) | 48 (15.89) | 82 (27.15) | 28 (9.27) |
| I am confident in making recommendations about starting osteoporosis medications (303) | 39 (12.87) | 153 (50.50) | 39 (12.87) | 52 (17.16) | 20 (6.60) |
| I am confident to counsel patients about osteoporosis medications (302) | 46 (15.23) | 157 (51.99) | 37 (12.25) | 48 (15.89) | 14 (4.64) |
| I am confident in making recommendations about osteoporosis medication treatment breaks (302) | 22 (7.28) | 86 (28.48) | 66 (21.85) | 102 (33.77) | 26 (8.61) |

## Supplementary Data 4 – Role Comparison of HCP Confidence in Delivering Osteoporosis Care (GP, Practice Nurse, Advanced Nurse Practitioner and Pharmacist)
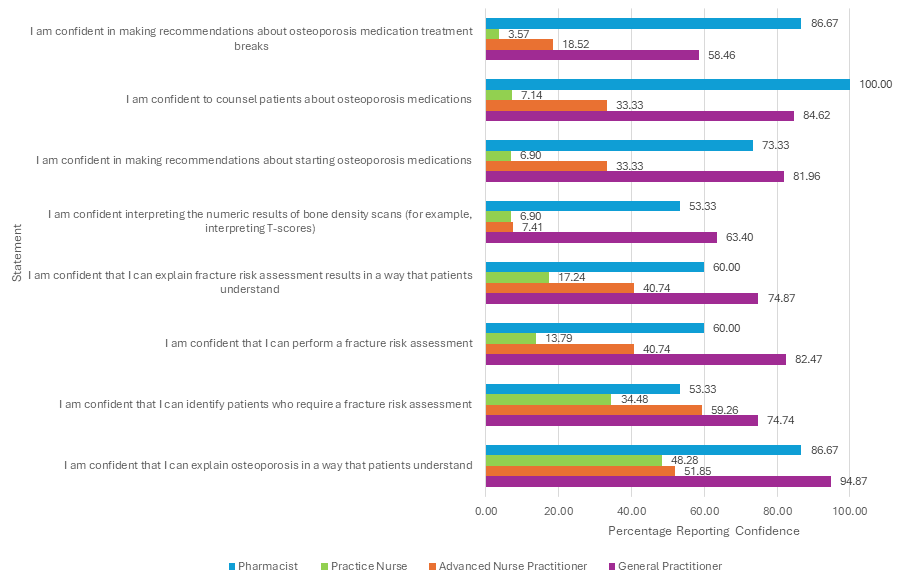


| **I am confident that I can explain osteoporosis in a way that patients understand** | | | |
| --- | --- | --- | --- |
| **Professional Role** | **Agree** | **Disagree** |  |
|  | **n (%)** | **n (%)** | **p-value** |
| General Practitioner | 185 (94.87) | 10 (5.13) | <0.001* |
| Practice Nurse | 14 (48.28) | 15 (51.72) |  |
| General Practitioner | 185 (94.87) | 10 (5.13) | <0.001* |
| Advanced Nurse Practitioner | 14 (51.85) | 13 (48.15) |  |
| General Practitioner | 185 (94.87) | 10 (5.13) | 0.207 |
| Pharmacist | 13 (86.67) | 2 (13.33) |  |
| Practice Nurse | 14 (48.28) | 15 (51.72) | 1 |
| Advanced Nurse Practitioner | 14 (51.85) | 13 (48.15) |  |
| Practice Nurse | 14 (48.28) | 15 (51.72) | 0.021* |
| Pharmacist | 13 (86.67) | 2 (13.33) |  |
| Advanced Nurse Practitioner | 14 (51.85) | 13 (48.15) | 0.042* |
| Pharmacist | 13 (86.67) | 2 (13.33) |  |

| **I am confident that I can identify patients who require a fracture risk assessment** | | | |
| --- | --- | --- | --- |
| **Professional Role** | **Agree** | **Disagree** |  |
|  | **n (%)** | **n (%)** | **p-value** |
| General Practitioner | 145 (74.74) | 49 (25.26) | <0.001* |
| Practice Nurse | 10 (34.48) | 19 (65.52) |  |
| General Practitioner | 145 (74.74) | 49 (25.26) | 0.107 |
| Advanced Nurse Practitioner | 16 (59.26) | 11 (40.74) |  |
| General Practitioner | 145 (74.74) | 49 (25.26) | 0.125 |
| Pharmacist | 8 (53.33) | 7 (46.67) |  |
| Practice Nurse | 10 (34.48) | 19 (65.52) | 0.107 |
| Advanced Nurse Practitioner | 16 (59.26) | 11 (40.74) |  |
| Practice Nurse | 10 (34.48) | 19 (65.52) | 0.334 |
| Pharmacist | 8 (53.33) | 7 (46.67) |  |
| Advanced Nurse Practitioner | 16 (59.26) | 11 (40.74) | 0.754 |
| Pharmacist | 8 (53.33) | 7 (46.67) |  |

| **I am confident that I can perform a fracture risk assessment** | | | |
| --- | --- | --- | --- |
| **Professional Role** | **Agree** | **Disagree** |  |
|  | **n (%)** | **n (%)** | **p-value** |
| General Practitioner | 160 (82.47) | 34 (17.53) | <0.001* |
| Practice Nurse | 4 (13.79) | 25 (86.21) |  |
| General Practitioner | 160 (82.47) | 34 (17.53) | <0.001* |
| Advanced Nurse Practitioner | 11 (40.74) | 16 (59.26) |  |
| General Practitioner | 160 (82.47) | 34 (17.53) | 0.044* |
| Pharmacist | 9 (60.00) | 6 (40.00) |  |
| Practice Nurse | 4 (13.79) | 25 (86.21) | 0.034* |
| Advanced Nurse Practitioner | 11 (40.74) | 16 (59.26) |  |
| Practice Nurse | 4 (13.79) | 25 (86.21) | 0.004* |
| Pharmacist | 9 (60.00) | 6 (40.00) |  |
| Advanced Nurse Practitioner | 11 (40.74) | 16 (59.26) | 0.336 |
| Pharmacist | 9 (60.00) | 6 (40.00) |  |

| **I am confident that I can explain fracture risk assessment results in a way that patients understand** | | | |
| --- | --- | --- | --- |
| **Professional Role** | **Agree** | **Disagree** |  |
|  | **n (%)** | **n (%)** | **p-value** |
| General Practitioner | 146 (74.87) | 49 (25.13) | <0.001* |
| Practice Nurse | 5 (17.24) | 24 (82.76) |  |
| General Practitioner | 146 (74.87) | 49 (25.13) | 0.001* |
| Advanced Nurse Practitioner | 11 (40.74) | 16 (59.26) |  |
| General Practitioner | 146 (74.87) | 49 (25.13) | 0.227 |
| Pharmacist | 9 (60.00) | 6 (40.00) |  |
| Practice Nurse | 5 (17.24) | 24 (82.76) | 0.076 |
| Advanced Nurse Practitioner | 11 (40.74) | 16 (59.26) |  |
| Practice Nurse | 5 (17.24) | 24 (82.76) | 0.007* |
| Pharmacist | 9 (60.00) | 6 (40.00) |  |
| Advanced Nurse Practitioner | 11 (40.74) | 16 (59.26) | 0.336 |
| Pharmacist | 9 (60.00) | 6 (40.00) |  |

| **I am confident interpreting the numeric results of bone density scans (for example, interpreting T-scores)** | | | |
| --- | --- | --- | --- |
| **Professional Role** | **Agree** | **Disagree** |  |
|  | **n (%)** | **n (%)** | **p-value** |
| General Practitioner | 123 (63.40) | 71 (36.60) | <0.001* |
| Practice Nurse | 2 (6.90) | 27 (93.10) |  |
| General Practitioner | 123 (63.40) | 71 (36.60) | <0.001* |
| Advanced Nurse Practitioner | 2 (7.41) | 25 (92.59) |  |
| General Practitioner | 123 (63.40) | 71 (36.60) | 0.581 |
| Pharmacist | 8 (53.33) | 7 (46.67) |  |
| Practice Nurse | 2 (6.90) | 27 (93.10) | 1 |
| Advanced Nurse Practitioner | 2 (7.41) | 25 (92.59) |  |
| Practice Nurse | 2 (6.90) | 27 (93.10) | 0.001* |
| Pharmacist | 8 (53.33) | 7 (46.67) |  |
| Advanced Nurse Practitioner | 2 (7.41) | 25 (92.59) | 0.002* |
| Pharmacist | 8 (53.33) | 7 (46.67) |  |

| **I am confident in making recommendations about starting osteoporosis medications** | | | |
| --- | --- | --- | --- |
| **Professional Role** | **Agree** | **Disagree** |  |
|  | **n (%)** | **n (%)** | **p-value** |
| General Practitioner | 159 (81.96) | 35 (18.04) | <0.001* |
| Practice Nurse | 2 (6.90) | 27 (93.10) |  |
| General Practitioner | 159 (81.96) | 35 (18.04) | <0.001* |
| Advanced Nurse Practitioner | 9 (33.33) | 18 (66.67) |  |
| General Practitioner | 159 (81.96) | 35 (18.04) | 0.498 |
| Pharmacist | 11 (73.33) | 4 (26.67) |  |
| Practice Nurse | 2 (6.90) | 27 (93.10) | 0.018* |
| Advanced Nurse Practitioner | 9 (33.33) | 18 (66.67) |  |
| Practice Nurse | 2 (6.90) | 27 (93.10) | <0.001* |
| Pharmacist | 11 (73.33) | 4 (26.67) |  |
| Advanced Nurse Practitioner | 9 (33.33) | 18 (66.67) | 0.023* |
| Pharmacist | 11 (73.33) | 4 (26.67) |  |

| **I am confident to counsel patients about osteoporosis medications** | | | |
| --- | --- | --- | --- |
| **Professional Role** | **Agree** | **Disagree** |  |
|  | **n (%)** | **n (%)** | **p-value** |
| General Practitioner | 165 (84.62) | 30 (15.38) | <0.001* |
| Practice Nurse | 2 (7.14) | 26 (92.86) |  |
| General Practitioner | 165 (84.62) | 30 (15.38) | <0.001* |
| Advanced Nurse Practitioner | 9 (33.33) | 18 (66.67) |  |
| General Practitioner | 165 (84.62) | 30 (15.38) | 0.136 |
| Pharmacist | 15 (100.00) | 0 (0.00) |  |
| Practice Nurse | 2 (7.14) | 26 (92.86) | 0.020* |
| Advanced Nurse Practitioner | 9 (33.33) | 18 (66.67) |  |
| Practice Nurse | 2 (7.14) | 26 (92.86) | <0.001* |
| Pharmacist | 15 (100.00) | 0 (0.00) |  |
| Advanced Nurse Practitioner | 9 (33.33) | 18 (66.67) | <0.001* |
| Pharmacist | 15 (100.00) | 0 (0.00) |  |

| **I am confident in making recommendations about osteoporosis medication treatment breaks** | | | |
| --- | --- | --- | --- |
| **Professional Role** | **Agree** | **Disagree** |  |
|  | **n (%)** | **n (%)** | **p-value** |
| General Practitioner | 81 (58.46) | 114 (41.54) | <0.001* |
| Practice Nurse | 1 (3.57) | 27 (96.43) |  |
| General Practitioner | 81 (58.46) | 114 (41.54) | <0.021* |
| Advanced Nurse Practitioner | 5 (18.52) | 22 (81.48) |  |
| General Practitioner | 81 (58.46) | 114 (41.54) | 0.001* |
| Pharmacist | 13 (86.67) | 2 (13.33) |  |
| Practice Nurse | 1 (3.57) | 27 (96.43) | 0.101 |
| Advanced Nurse Practitioner | 5 (18.52) | 22 (81.48) |  |
| Practice Nurse | 1 (3.57) | 27 (96.43) | <0.001* |
| Pharmacist | 13 (86.67) | 2 (13.33) |  |
| Advanced Nurse Practitioner | 5 (18.52) | 22 (81.48) | <0.001* |
| Pharmacist | 13 (86.67) | 2 (13.33) |  |

## Supplementary Data 5 – Self Reported Roles Compared to Role Perceived by Others

### GPs

| **Role** | GP (self-reported, n=191) | | | | GP (perceived, n=123) | | | |
| --- | --- | --- | --- | --- | --- | --- | --- | --- |
|  | Yes | No | Do not know | No response | Yes | No | Do not know | No response |
|  | n (%) | n (%) | n (%) | n (%) | n (%) | n (%) | n (%) | n (%) |
| Osteoporosis case finding | 164 (85.86) | 17 (8.90) | 8 (4.19) | 2 (1.05) | 89 (72.36) | 10 (8.13) | 19 (15.45) | 5 (4.07) |
| Assessment of fracture risk | 183 (95.81) | 5 (2.62) | 2 (1.05) | 1 (0.52) | 100 (81.30) | 5 (4.07) | 16 (13.01) | 2 (1.63) |
| Assessment of falls risk | 166 (86.91) | 21 (10.99) | 3 (1.57) | 1 (0.52) | 94 (76.42) | 11 (8.94) | 16 (13.01) | 2 (1.63) |
| Referring for bone density scans | 188 (98.43) | 0 (0.00) | 2 (1.05) | 1 (0.52) | 115 (93.50) | 1 (0.81) | 6 (4.88) | 1 (0.81) |
| Requesting blood tests to exclude secondary causes of osteoporosis | 186 (97.38) | 3 (1.57) | 2 (1.05) | 0 (0.00) | 114 (92.68) | 1 (0.81) | 7 (5.69) | 1 (0.81) |
| Osteoporosis medication counselling | 189 (98.95) | 1 (0.52) | 1 (0.52) | 0 (0.00) | 102 (82.93) | 2 (1.63) | 18 (14.63) | 1 (0.81) |
| Osteoporosis medication prescribing | 190 (99.48) | 0 (0.00) | 1 (0.52) | 0 (0.00) | 114 (92.68) | 1 (0.81) | 7 (5.69) | 1 (0.81) |
| Osteoporosis medication reviews (e.g. after five years of treatment) | 177 (92.67) | 9 (4.71) | 4 (2.09) | 1 (0.52) | 92 (74.80) | 8 (6.50) | 22 (17.89) | 1 (0.81) |
| Referral to secondary care for injectable therapy | 184 (96.34) | 5 (2.62) | 2 (1.05) | 0 (0.00) | 99 (80.49) | 1 (0.81) | 22 (17.89) | 1 (0.81) |
| Referral to secondary care for expert opinion | 186 (97.38) | 2 (1.05) | 1 (0.52) | 2 (1.05) | 114 (92.68) | 0 (0.00) | 8 (6.50) | 1 (0.81) |

### Advanced Nurse Practitioners (ANPs)

| Role | ANP (reported, n=17) | | | | ANP (perceived, n=136) | | | |
| --- | --- | --- | --- | --- | --- | --- | --- | --- |
|  | Yes | No | Do not Know | No response | Yes | No | Do not Know | No response |
|  | n (%) | n (%) | n (%) | n (%) | n (%) | n (%) | n (%) | n (%) |
| Osteoporosis case finding | 11 (64.71) | 3 (17.65) | 2 (11.76) | 1 (5.88) | 86 (63.24) | 26 (19.12) | 19 (13.97) | 5 (3.68) |
| Assessment of fracture risk | 15 (88.24) | 2 (11.76) | 0 (0.00) | 0 (0.00) | 82 (60.29) | 28 (20.59) | 22 (16.18) | 4 (2.94) |
| Assessment of falls risk | 17 (100.00) | 0 (0.00) | 0 (0.00) | 0 (0.00) | 97 (71.32) | 16 (11.76) | 18 (13.24) | 5 (3.68) |
| Referring for bone density scans | 11 (64.71) | 6 (35.29) | 0 (0.00) | 0 (0.00) | 74 (54.41) | 41 (30.15) | 16 (11.76) | 5 (3.68) |
| Requesting blood tests to exclude secondary causes of osteoporosis | 17 (100.00) | 0 (0.00) | 0 (0.00) | 0 (0.00) | 93 (68.38) | 21 (15.44) | 17 (12.50) | 5 (3.68) |
| Osteoporosis medication counselling | 14 (82.35) | 2 (11.76) | 1 (5.88) | 0 (0.00) | 72 (52.94) | 43 (31.62) | 17 (12.50) | 4 (2.94) |
| Osteoporosis medication prescribing | 14 (82.35) | 3 (17.65) | 0 (0.00) | 0 (0.00) | 69 (50.74) | 48 (35.29) | 15 (11.03) | 4 (2.94) |
| Osteoporosis medication reviews (e.g. after five years of treatment) | 12 (70.59) | 4 (23.53) | 1 (5.88) | 0 (0.00) | 52 (38.24) | 53 (38.97) | 24 (17.65) | 7 (5.15) |
| Referral to secondary care for injectable therapy | 14 (82.35) | 2 (11.76) | 1 (5.88) | 0 (0.00) | 60 (44.12) | 50 (36.76) | 19 (13.97) | 7 (5.15) |
| Referral to secondary care for expert opinion | 15 (88.24) | 2 (11.76) | 0 (0.00) | 0 (0.00) | 68 (50.00) | 42 (30.88) | 19 (13.97) | 7 (5.15) |

### Practice Nurses

| Role | Practice Nurse (reported, n=16) | | |  | Practice Nurse (perceived, n=104) | | |  |
| --- | --- | --- | --- | --- | --- | --- | --- | --- |
|  | Yes | No | Do not Know | No response | Yes | No | Do not Know | No response |
|  | n (%) | n (%) | n (%) | n (%) | n (%) | n (%) | n (%) | n (%) |
| Osteoporosis case finding | 4 (25.00) | 11 (68.75) | 1 (6.25) | 0 (0.00) | 30 (28.85) | 54 (51.92) | 11 (10.58) | 9 (8.65) |
| Assessment of fracture risk | 4 (25.00) | 11 (68.75) | 1 (6.25) | 0 (0.00) | 34 (32.69) | 51 (49.04) | 11 (10.58) | 8 (7.69) |
| Assessment of falls risk | 10 (62.50) | 6 (37.50) | 0 (0.00) | 0 (0.00) | 59 (56.73) | 33 (31.73) | 7 (6.73) | 5 (4.81) |
| Referring for bone density scans | 1 (6.25) | 14 (87.50) | 0 (0.00) | 1 (5.88) | 5 (4.81) | 81 (77.88) | 7 (6.73) | 11 (10.58) |
| Requesting blood tests to exclude secondary causes of osteoporosis | 3 (18.75) | 12 (75.00) | 1 (6.25) | 0 (0.00) | 19 (18.27) | 69 (66.35) | 6 (5.77) | 10 (9.62) |
| Osteoporosis medication counselling | 4 (25.00) | 11 (68.75) | 1 (6.25) | 0 (0.00) | 20 (19.23) | 67 (64.42) | 9 (8.65) | 8 (7.69) |
| Osteoporosis medication prescribing | 1 (6.25) | 15 (93.75) | 0 (0.00) | 0 (0.00) | 5 (4.81) | 80 (76.92) | 9 (8.65) | 10 (9.62) |
| Osteoporosis medication reviews (e.g. after five years of treatment) | 2 (12.50) | 14 (87.50) | 0 (0.00) | 0 (0.00) | 11 (10.58) | 76 (73.08) | 9 (8.65) | 8 (7.69) |
| Referral to secondary care for injectable therapy | 0 (0.00) | 16 (100.00) | 0 (0.00) | 0 (0.00) | 1 (0.96) | 85 (81.73) | 8 (7.69) | 10 (9.62) |
| Referral to secondary care for expert opinion | 1 (6.25) | 15 (93.75) | 0 (0.00) | 0 (0.00) | 4 (3.85) | 83 (79.81) | 7 (6.73) | 10 (9.62) |

### Pharmacists

| **Role** | Pharmacist (reported, n=14) | | | | Pharmacist (perceived, n=227) | | | |
| --- | --- | --- | --- | --- | --- | --- | --- | --- |
|  | Yes | No | Do not Know | No response | Yes | No | Do not Know | No response |
|  | n (%) | n (%) | n (%) | n (%) | n (%) | n (%) | n (%) | n (%) |
| Osteoporosis case finding | 7 (50.00) | 5 (35.71) | 1 (7.14) | 1 (7.14) | 58 (25.55) | 129 (56.83) | 31 (13.66) | 9 (3.96) |
| Assessment of fracture risk | 10 (71.43) | 3 (21.43) | 1 (7.14) | 0 (0.00) | 66 (29.07) | 119 (52.52) | 31 (13.66) | 11 (4.85) |
| Assessment of falls risk | 7 (50.00) | 6 (42.86) | 1 (7.14) | 0 (0.00) | 41 (18.06) | 147 (64.76) | 26 (11.45) | 13 (5.73) |
| Referring for bone density scans | 10 (71.43) | 3 (21.43) | 1 (7.14) | 0 (0.00) | 15 (6.61) | 173 (76.21) | 25 (11.01) | 14 (6.17) |
| Requesting blood tests to exclude secondary causes of osteoporosis | 7 (50.00) | 6 (42.86) | 1 (7.14) | 0 (0.00) | 54 (23.79) | 141 (62.11) | 18 (7.93) | 14 (6.17) |
| Osteoporosis medication counselling | 14 (100.00) | 0 (0.00) | 0 (0.00) | 0 (0.00) | 198 (87.22) | 12 (5.29) | 12 (5.29) | 5 (2.20) |
| Osteoporosis medication prescribing | 14 (100.00) | 0 (0.00) | 0 (0.00) | 0 (0.00) | 153 (67.40) | 50 (22.03) | 16 (7.05) | 8 (3.52) |
| Osteoporosis medication reviews (e.g. after five years of treatment) | 14 (100.00) | 0 (0.00) | 0 (0.00) | 0 (0.00) | 183 (80.62) | 24 (10.57) | 17 (7.49) | 3 (1.32) |
| Referral to secondary care for injectable therapy | 9 (64.29) | 4 (28.57) | 1 (7.14) | 0 (0.00) | 26 (11.45) | 160 (70.48) | 26 (11.45) | 15 (6.61) |
| Referral to secondary care for expert opinion | 10 (71.43) | 3 (21.43) | 1 (7.14) | 0 (0.00) | 27 (11.89) | 160 (70.48) | 24 (10.57) | 16 (7.05) |

## Supplementary Data 6 – Primary Care Case Finding Systems

| Does your practice: | Yes, the practice does | No, the practice does not | Do not know | No answer |
| --- | --- | --- | --- | --- |
|  | n (%) | n (%) | n (%) | n (%) |
| have a system (or mechanism) to identify all patients who have sustained a fragility fracture for the purposes of fracture risk assessment? | 133 (42.90) | 69 (22.26) | 107 (34.52) | 1 (0.32) |
| invite all patients with a history of fragility fracture for a fracture risk assessment? | 87 (28.06) | 98 (31.61) | 125 (40.32) | 0 (0.00) |
| have a system (or mechanism) to identify all patients who use systemic glucocorticoids for the purposes of fracture risk assessment? | 108 (34.84) | 86 (27.74) | 116 (37.42) | 0 (0.00) |
| invite patients who use systemic glucocorticoids for a fracture risk assessment? | 69 (22.26) | 97 (31.29) | 138 (44.52) | 6 (1.94) |
| have a system (or mechanism) to identify all patients over the age of 50 years who have a history of falls for the purposes of fracture risk assessment? | 72 (23.23) | 111 (35.81) | 122 (39.35) | 5 (1.61) |
| invite patients with a history of falls for a fracture risk assessment? | 64 (20.65) | 126 (40.65) | 117 (37.74) | 3 (0.97) |
| offer all patients prescribed osteoporosis medications a medication review? | 212 (68.39) | 35 (11.29) | 61 (19.68) | 2 (0.65) |
| have a system to identify people who have been taking oral bisphosphonates for five or ten years with the purpose of arranging a treatment review? | 138 (44.52) | 67 (21.61) | 103 (33.23) | 2 (0.65) |
| n=310, HCPs (n=283) and non-HCPs (n=27) | | | | |
